# Neural correlates of gentle skin stroking in early infancy

**DOI:** 10.1016/j.dcn.2017.10.004

**Published:** 2017-10-24

**Authors:** Jetro J. Tuulari, Noora M. Scheinin, Satu Lehtola, Harri Merisaari, Jani Saunavaara, Riitta Parkkola, Isac Sehlstedt, Linnea Karlsson, Hasse Karlsson, Malin Björnsdotter

**Affiliations:** a FinnBrain Birth Cohort Study, Turku Brain and Mind Center, Institute of Clinical Medicine, University of Turku, Turku, Finland; b Turku PET Centre, University of Turku, Turku, Finland; c Department of Psychiatry, University of Turku and Turku University Hospital, Turku, Finland; d Department of Medical Physics, Turku University Hospital, Turku, Finland; e Department of Radiology, University of Turku and Turku University Hospital, Turku, Finland; f Center for Social and Affective Neuroscience, Linköping University, Sweden; g Department of Child Psychiatry, University of Turku and Turku University Hospital, Turku, Finland; h Institute of Neuroscience and Physiology, University of Gothenburg, Sweden; i Department of Psychology, University of Gothenburg, Sweden

**Keywords:** Infants, Development, fMRI, Touch, Social touch

## Abstract

Physical expressions of affection play a foundational role in early brain development, but the neural correlates of affective touch processing in infancy remain unclear. We examined brain responses to gentle skin stroking, a type of tactile stimulus associated with affectionate touch, in young infants. Thirteen term-born infants aged 11–36 days, recruited through the FinnBrain Birth Cohort Study, were included in the study. Soft brush strokes, which activate brain regions linked to somatosensory as well as socio-affective processing in children and adults, were applied to the skin of the right leg during functional magnetic resonance imaging. We examined infant brain responses in two regions-of-interest (ROIs) known to process gentle skin stroking – the postcentral gyrus and posterior insular cortex – and found significant responses in both ROIs. These results suggest that the neonate brain is responsive to gentle skin stroking within the first weeks of age, and that regions linked to primary somatosensory as well as socio-affective processing are activated. Our findings support the notion that social touch may play an important role in early life sensory processing. Future research will elucidate the significance of these findings for human brain development.

## Highlights

• The infant brain is sensitive to gentle skin stroking within the first weeks of age.
• The postcentral gyrus and posterior insular cortex are responsive to stroking.
• Social touch activates both somatosensory and socio-affective brain areas in infancy.

## 1 Introduction

Animal research emphasizes the pervasive effects of early physical contact on healthy nervous system development and behavior across the lifespan (Koch et al., 2012, Kolb et al., 2012). For instance, caring maternal behavior, such as licking and grooming, profoundly impacts life-long behavioral trajectories through epigenetic effects on brain morphology (Bagot et al., 2012, Suderman et al., 2012, Zhang and Meaney, 2010). Indeed, sensory experiences are critical for healthy maturation of neural circuits and the rodent neonate central nervous system is exquisitely sensitive to light touch (Koch and Fitzgerald, 2013). In humans, parental touch plays a crucial role during development (Cascio, 2010, Corbetta and Snapp-Childs, 2009, Feldman et al., 2010), and expressions of physical affection during early stages lay the foundation for the development of socio-emotional wellbeing throughout life (Walker and McGlone, 2013, Field, 2010). However, the neural correlates of socio-affective touch processing in infancy are surprisingly poorly understood. The aim of this study was therefore to examine the neural correlates of social touch in infancy. Specifically, we examined caress-like, gentle skin stroking, a type of tactile stimulation intimately associated with social interaction and affectionate touch (Croy et al., 2016).

In the mature nervous system, skin stroking vigorously activates low-threshold mechanoreceptors and Aβ afferents which rapidly convey sensory information to the somatosensory cortices (Abraira and Ginty, 2013). Aβ afferents innervate the entire body, including both hairy and glabrous skin (Goodwin and Wheat, 2008), and play a critical role in coding sensory-discriminative dimensions of touch and movement (McGlone et al., 2014). Although Aβ afferents are immature at birth and are thought to function poorly in infancy (Ferrington and Rowe, 1980, Ferrington et al., 1984), the system still signals tactile stimuli at an early age: palm stimulation activates preterm and term born infants’ postcentral gyrus (Arichi et al., 2012, Souweidane et al., 1999), and application of tactile stimuli to the plantar surface of the foot of two-week-old infants yields activations in primary sensory areas (Williams et al., 2015). In light of these findings, the first objective of this study was to determine whether somatosensory regions respond also to gentle skin stroking of the hairy skin in early infancy. In particular, gentle stroking of the hairy skin elicits responses in the primary somatosensory cortex contralateral to the stimulus in children and adults alike (Björnsdotter et al., 2014), and we therefore hypothesized that this region would respond also in infants.

In addition to the Aβ-somatosensory system, a specific class of unmyelinated C-tactile (CT) afferents are activated by gentle skin stroking of the hairy, but not glabrous, skin in adults (Morrison, 2012). CT afferents belong to the group of unmyelinated C fibers that carry basic physiological signals such as pain and temperature. These are known to mature early: painful stimuli evoke neural responses already at 25 weeks of gestation (Slater et al., 2006), and at 35–37 weeks, brain responses as measured with electroencephalography (EEG) are similar to those seen in adults (Fabrizi et al., 2011). The specific function of the CT system is largely unknown, but the fibers respond selectively to pleasant, caress-like gentle skin stroking of the hairy skin (Löken et al., 2009, Vallbo et al., 1993) and the system is hypothesized to play a role in encoding socio-affective dimensions of touch (McGlone et al., 2014). The primary cortical target for CT afferents is thought to be the posterior insular cortex (Björnsdotter et al., 2009, Olausson et al., 2002, Olausson et al., 2008), which is associated with socio-affective tactile processing in adults (Morrison et al., 2011a, Morrison et al., 2011b). The development of insular sensitivity to gentle skin stroking is exceptionally poorly understood, however. The second objective of this study was therefore to examine whether the infant insular cortex responds to gentle skin stroking. Specifically, gentle stroking of the hairy skin elicits responses in the posterior portion of the insular cortex contralateral to the stimulus in children as young as four years old (Björnsdotter et al., 2014), and we therefore hypothesized that this region would respond also in infancy.

Finally, gentle skin stroking elicits responses across a wide range of brain regions beyond the primary somatosensory and insular cortices that may be of potential importance for development (Björnsdotter et al., 2014, Gordon et al., 2013). We therefore also conducted an exploratory whole-brain analysis to search for additional activations outside the *a priori* defined regions of interest.

## 2 Material and methods

The study was conducted in accordance with the Declaration of Helsinki, and it was approved by the Joint Ethics Committee of the University of Turku and the Hospital District of Southwest Finland.

### 2.1 Participants and recruitment

During the period of 05–08/2015, infants born to families taking part in the FinnBrain Birth Cohort Study (finnbrain.fi) were recruited for functional MRI studies. When the infants were aged 2–5 weeks, each family was personally contacted and recruited into the present study via telephone calls (by author SL). Exclusion criteria for the infants were: occurrence of any perinatal complications with potential neurological consequences (e.g. hypoxia), less than 5 points in the 5 min Apgar score, previously diagnosed central nervous system anomaly or an abnormal finding in a previous MRI scan (with clinical indications), delivery at less than 32 weeks of pregnancy or birth weight less than 1500 g (these criteria were confirmed through a structured interview over the telephone).

Families were provided oral and written information about the study, and the parents provided written consent to participate on behalf of their baby.

### 2.2 Tactile stimuli and experimental protocol

During MRI acquisition, a trained experimenter (author JJT) manually applied gentle brush strokes to infants’ right anterior shin region (along the tibia) in a proximal to distal direction. This site was chosen due to ease of access, as the babies were wrapped in vacuum mattress that blocked upper extremities. Also, the investigator leaned on the scanner bed inside the scanner bore, without touching the infants, whilst delivering the stimuli. The length of the stimulated area was measured to cover 15 cm, and brush strokes were applied at a velocity of 3 cm/s for 15s, with randomized inter-stimulus intervals of 10–15 s (resulting in 3 strokes in one 15 s block). The experimenter was guided by auditory cues delivered through the scanner head phones. A total of 11 stimuli were applied.

### 2.3 MRI scanning visits

At the scanning site (Medical Imaging Centre of Hospital District of Southwest Finland), the families were received by a trained and experienced radiographer and the researchers. Before the scan, the scanning protocol was revised with the parents and the absence of safety risks (e.g. pacemakers, inner ear implants, other metal parts) was confirmed by the personnel. The infants were then fed with (breast)milk to get them to sleep and subsequently gently swaddled into a vacuum mattress. No anesthetics were used. All the scans took place in the afternoon to early evening hours (16:30–20:00).

All infants were provided with hearing protection (wax plugs and custom-sized ear muffs). Standard ear muffs were given to parents, as they stayed in the scanning room throughout the whole scanning session. The scanning was observed by the personnel from the control room through a window with a microphone contact to a parent and a loudspeaker sending the sounds from the scanning room allowing the staff to hear if the infant should have woken up. If the baby did not fall asleep before or during the scan, the session was ended. After the scan, the family was given a small present (a bop hat or a body suit) as a thank you for their participation.

Each set of structural infant images was checked by an experienced neuroradiologist (author RP) to detect any possible pathological changes visible in the scans. In the case of a pathological finding, the parents were referred to a child neurologist and a neurological check-up at ages 6–8 months. In the current sample, one participant had incidental findings (minor hemorrhages) that were deemed minor by the radiologist and assured to be outside the cerebral tissues (not confounding the analysis). Also, this infant did not exhibit developmental problems at the check-up. Radiology reports were delivered to the researchers who then communicated them to the family within 1–4 weeks of the scans.

### 2.4 MRI acquisition

MRI scans were conducted on a Siemens Magnetom Verio 3T scanner (Siemens Medical Solutions, Erlangen, Germany). A 12-element Head Matrix coil allowed the use of Generalized Autocalibrating Partially Parallel Acquisition (GRAPPA) technique to accelerate acquisitions (PAT factor of 2 was used). Sequence parameters of the 2D Dual Echo TSE (Turbo Spin Echo) sequence were optimized so that “whisper” gradient mode could be used in order to reduce acoustic noise during the scan. Slice thickness was 1 mm in order to acquire isotropic 1.0 × 1.0 × 1.0 mm voxels. TR time of 12070 ms and effective TE times of 13 ms and 102 ms were used to produce both PD-weighted and T2-weighted images from the same acquisition. The total number of slices was 128. T1-weighted 3D MPRAGE (Magnetization Prepared Rapid Acquisition Gradient Echo) sequence with isotropic 1.0 × 1.0 × 1.0 mm voxels was used for anatomical imaging as well. The sequences included DTI imaging (not reported here). Functional MRI consisted of 120 volumes with voxel size 3.0 × 3.0 × 3.0 mm, TR 3000 ms, TE 30 ms, flip angle of 80 ° and 42 axial slices without gaps. Prior to fMRI acquisition, all infants had slept during the 45–50 min required for structural scanning. The total duration of the complete scanning protocol did not exceed 60 min.

### 2.5 Data pre-processing and statistical analyses

Preprocessing and statistical analyses were conducted using SPM12 (http://www.fil.ion.ucl.ac.uk/spm/software/spm12/). Functional data preprocessing included slice time correction, realignment to the first volume of the first run, and normalized to the University of North Carolina at Chapel Hill neonate atlas (Shi et al., 2011). Motion artifacts were examined using the Artifact Detection Toolbox (ART) (https://www.nitrc.org/projects/artifact_detect/). Volumes where global signal deviated more than two standard deviations from the mean signal or where the difference in motion between two neighboring volumes exceeded 1 mm were identified as outlier volumes.

The stimuli were modeled as one predictor convolved with the standard SPM12 hemodynamic response function. A fixed effects general linear model analysis, including motion parameters and outlier volumes as regressors of no interest, was performed in each individual infant.

**Fig. 1:**
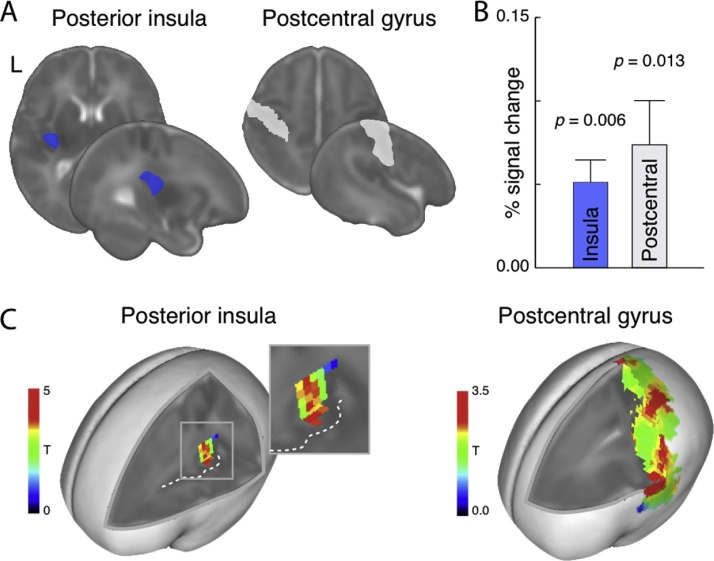
Brain responses to skin stroking in insular and postcentral regions of interest. (A) Left hemisphere insular and postcentral gyrus regions of interest (ROIs) from the University of North Carolina at Chapel Hill neonate Automated Anatomical Labeling atlas. (B) Average percent signal change in response to skin stroking extracted from the ROIs. Error bars indicate standard error of the mean. Significance of planned comparisons (one-sample Wilcoxon signed rank tests) are indicated. L = left hemisphere. (C) Post-hoc voxel-wise analysis results.

In order to test the hypotheses that gentle skin stroking elicits responses in the primary somatosensory and posterior insular cortex contralateral to the stimulus, a region-of-interest (ROI) approach was applied. Here, the left postcentral gyrus, the anatomical location of the primary somatosensory cortex, and left insular cortex were obtained from the University of North Carolina at Chapel Hill neonate Automated Anatomical Labeling (AAL) atlas. As we were specifically interested in the posterior insular cortex due to its role as a primary projection target of CT afferents (Björnsdotter et al., 2009), the insular AAL region was divided into three equal portions across the axial dimension and the two anterior portions were discarded (Fig. 1A). Individual infant percent signal change in response to skin stroking were then extracted from each ROI using the MarsBar toolbox (http://marsbar.sourceforge.net/), and we then tested whether these were significantly larger than zero using non-parametric one-sample Wilcoxon signed rank tests. In a *post hoc* analysis, we also examined the spatial distribution of the results by conducting a voxel-wise analysis within the ROIs. Here, we identified all local maxima more than 8 mm apart, as per the default SPM12 settings.

In order to examine brain responses outside the *a priori* defined regions of interest, we conducted an exploratory whole-brain analysis. The results were assessed using non-parametric permutation testing clusterwise inference as implemented in the Statistical nonParametric Mapping toolbox (http://warwick.ac.uk/snpm), with a cluster-forming threshold of 0.005 and a family-wise error (FWE) of 0.05. In order to simplify future hypothesis generation and later meta-analyses, we also reported any results passing the cluster-forming threshold of p < 0.005.

## 3 Results

### 3.1 Participants and motion

Parents of 13 infants volunteered for their child to participate in the study. Excessive motion in three infants rendered the data unusable, leaving data from 10 infants for analysis. Demographic data of these infants are presented in Table 1.

**Table 1:** Demographics of infants included in the analysis (N = 10).

	Median	Range
Maternal age (years)	30.2	19.1–37.4
Maternal BMI	26.0	21.0–34.4
Paternal age (years)	29.4	20.0–38.6
Gestational weeks at birth	39.6	39.0–41.1
Age at scan from term (d)	21.5	15–29
Age at scan from birth (d)	25.0	13–31
Birth weight (g)	3544	3085–4050
Birth height (cm)	51.5	48.0–54.0
Head circumference (cm)	35.3	34.5–37.5

In the final infant group analysis, the mean number of volumes discarded from analysis due to motion was 16.8 (standard deviation 12.3, range 0–36; out of the total 120 volumes).

### 3.2 Region of interest analysis

Infants’ brain responses were significantly larger than zero in both the postcentral gyrus *(p* = 0.013) and the insular cortex (*p* = 0.006) (Fig. 1). There were no significant correlations with age from birth or age from term in infants in any ROI (Spearman's rank correlation, all *p >* 0.15).

The *post hoc* voxel-wise analysis showed that the insular ROI contained one peak response (T = 4.79, *z* = 3.29) located in the anterior and ventral portion of the ROI (Fig. 1C). The postcentral gyrus contained a number of peaks along the dorsal – ventral axis, including a very dorsal area which may correspond to the primary somatosensory leg cortex (T = 2.96, z = 2.41). Also, a peak was found in the ventral region of the ROI that may correspond to the secondary somatosensory cortex (T = 3.22, *z* = 2.56).

### 3.3 Whole-brain results

**Fig. 2:**
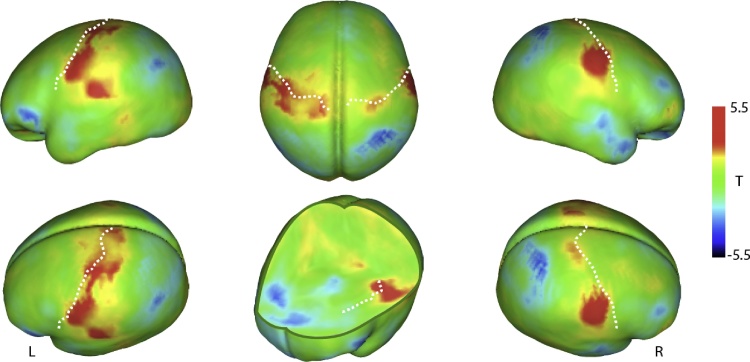
Whole-brain responses to skin stroking in infants. The results are rendered on the University of North Carolina at Chapel Hill neonate template brain, and show unthresholded voxel-wise T statistics. Dotted lines indicate the central sulcus and insular cortex. L = left hemisphere, R = right hemisphere.

The exploratory whole-brain analysis did not reveal any additional significant responses that survived correction for multiple comparisons at pFWE < 0.05. Several regions exhibited activations at the cluster-forming threshold of p < 0.005, however, including the inferior parietal, parahippocampal, dorsal and ventral right postcentral gyrus, and superior temporal cortex (Table 2). Uncorrected whole-brain analysis results are shown in Fig. 2.

**Table 2:** Brain responses to skin stroking in infants.

Region		x	y	z	T	Nr. Voxels
Inferior Parietal	Left	−24	−32	32	3.13	9
	Left	−36	−22	32	2.94	1
	Left	−40	−22	32	2.93	3
Parahippocampal	Right	14	−16	−18	3.45	11
Postcentral	Right	42	−6	20	3.63	42
Superior Temporal	Left	−38	−8	8	3.09	7
	Left	−40	−6	24	3.48	176

The results show peak activation results at uncorrected p < 0.005, and the indicated regions refer to the infant Automated Anatomical Labeling (AAL) atlas. Coordinates refer to the University of North Carolina at Chapel Hill neonate atlas.

## 4 Discussion

We examined brain responses to gentle skin stroking in early infancy, and found significant activations in the postcentral gyrus and posterior insular cortex.

The finding of significant postcentral gyrus responses to skin stroking is corroborated by previous findings of somatosensory cortex activity in early infancy (Arichi et al., 2012, Souweidane et al., 1999, Williams et al., 2015, Allievi et al., 2016). However, previous studies applied sensory-discriminatory types of somatosensory stimuli, such as rubbing of the hand (Souweidane et al., 1999) or passive movements (Allievi et al., 2016). The finding that tactile stimuli of a social character also activates the somatosensory cortex in early infancy therefore highlights the putative importance of interpersonal touch for brain development. Additionally, we applied the stimuli to the hairy skin, which is innervated by a different set of mechano-sensitive neurons than the glabrous skin of the palm and the soles of the feet (Abraira and Ginty, 2013). The hairy skin presents the largest area of contact between caregivers and young infants, and our results may have therefore have particular implications for early life interventions such as skin-to-skin contact in preterm infants (Feldman et al., 2014). Taken together, the demonstration of postcentral gyrus responses to gentle stroking of the hairy skin in early infancy underscores the potentially important role of social touch in early life sensory processing.

The postcentral gyrus AAL mask was relatively large and covered the full extent of the postcentral gyrus; the identified effect in this region may therefore stem from either the primary or secondary somatosensory cortices, or a combination of both. However, the *post hoc* voxel-wise analysis revealed peaks in the ventral and dorsal postcentral gyrus, suggesting that both the secondary and primary somatosensory cortex likely contributed to the effect. Similar to what is typically found in adults (Davidovic et al., 2016), responses were also observed in the right postcentral gyrus in infants, suggesting that a bilateral representation of tactile stimuli may be present in the somatosensory cortex at an early age. Moreover, the activations were distributed across the ventral-dorsal axis of the postcentral gyrus, resembling the pattern of skin stroking responses observed in adults (Davidovic et al., 2016).

We also found that the posterior insular cortex responded to gentle skin stroking in infants. The posterior insular cortex region is considered the primary cortical target for CT fibers in adults (Björnsdotter et al., 2009), and this finding is therefore consistent with a functional CT system in early infancy. However, since the insular cortex may also respond to stroking of the glabrous skin (Williams et al., 2015, Gordon et al., 2013), which is not innervated by CT afferents, possible signaling through myelinated afferents cannot be ruled out. Also, we could not assess behavioral responses to establish whether the infant’s perceived the skin stroking as pleasurable, as is found in adults (Löken et al., 2009, Davidovic et al., 2016, Kaiser et al., 2015, Bennett et al., 2013) and children (Sehlstedt et al., 2016). However, infants as young as nine months are selectively sensitive to gentle skin stroking (Fairhurst et al., 2014), indicating that the neural substrate required for detecting pleasurable caresses develops early. Our study suggests that the posterior insular cortex may constitute such a substrate, opening the possibility that skin stroking could be selectively processed already in early infancy. Further research examining a range of different tactile stimuli, including types to which CT afferents respond poorly, such as vibration or fast brush strokes, is required to determine when and how this sensitivity develops. Such studies may also detect when the nervous system is sufficiently mature to distinguish socio-affective and sensory-discriminative tactile stimuli, similar to the developmental threshold at which painful and non-painful stimuli become differentiated at 35–37 weeks of gestation (Fabrizi et al., 2011).

The finding that the insular cortex responds to skin stroking in infants has particular implications for neonatal care. Specifically, a recent study by Maitre et al. (2017) showed that repeated painful experiences in premature infants attenuate somatosensory responses to light touch, whereas supportive tactile experiences increased responses amplitudes (Maitre et al. 2017). The authors propose that cross-modal effects may be a driving mechanism behind this phenomenon. Given the known but enigmatic role of the CT system and posterior insular cortex in pain processing (Liljencrantz et al. 2013), our results suggest that the CT – insula circuit could play an important role in this development. Further studies assessing effects of gentle skin stroking on infant bran maturation are therefore urgent.

The exploratory whole-brain analysis did not reveal any additional significantly activated brain regions. However, at an uncorrected threshold, we found responses in parietal cortex regions and superior temporal areas. Putative superior temporal activations may be of particular interest in future studies of socio-emotional development and attachment, as this region is linked to individual differences in social perception (Björnsdotter et al., 2016) including perceived pleasantness of skin stroking (Davidovic et al., 2016). However, as these results did not pass correction for multiple comparisons they should be interpreted with caution. Further studies in a larger number of infants are required to verify activity in these regions.

There are a number of limitations of this study. First, it is unclear if and how sleep affects brain processing of tactile stimuli; sedation is, for example, known to attenuate sensory response in infants (Williams et al., 2015). Nevertheless, our study supports previous findings showing detectable responses to a range of sensory stimuli in sleeping infants (Williams et al., 2015, Graham et al., 2015). Second, our sample size was relatively small, although within the range of previously published fMRI activation studies (Graham et al. 2015). Third, motion is a key challenge in infant fMRI (Graham et al. 2015). Nevertheless, motion is unlikely to have contributed substantially to the observed effect in this study: data in ten out of thirteen infants were of sufficient quality for analysis, and only 14% of the fMRI volumes were discarded on average due to motion. Although remaining micro-movements may be an issue (Graham et al. 2015), the simplicity of the stimulation protocol and the robustness of the results suggest that these should are unlikely to have had any major effect on the reported results. Fourth, the manual application of the stimuli added a source of uncontrolled variability within and between participants. However, manual application dominates fMRI studies of affective touch in adults (Björnsdotter et al. 2014; Björnsdotter et al. 2009; Davidovic et al. 2016; Morrison et al. 2011a; Olausson et al. 2008; Morrison et al. 2011b; Gordon et al. 2013; Kaiser et al. 2015), and our results should therefore be comparable to previous findings. Fifth, we used an echo time of 30 ms, whereas recent research in infant neuroimaging shows that longer echo times (∼50 ms) substantially improve sensitivity (Goksan et al. 2016). Sixth, given the highly limited fMRI time allowed by the Ethics Committee (6 min) in combination with the high risk of data loss due to motion, we opted for collection of robust main effect of slow skin stroking with no control condition. It is therefore unclear which particular aspect of the stimuli elicited the observed responses. Further studies including control conditions such as vibration (Davidovic et al., 2016) or fast stroking (Morrison et al., 2011a) are needed to determine whether the observed effects are selective to slow skin stroking. Finally, the current sample is cross-sectional and does not address brain development *per se* (Karmiloff-Smith, 2010); future follow-up studies within the FinnBrain Birth Cohort Study may allow for clarification of the maturation of sensory processing in further detail, as well as its practical implications for child development.

## 5 Conclusions

Our results suggest that the neonate brain is responsive to gentle skin stroking already within the first weeks of age, and that regions linked to somatosensory as well as socio-affective processing are activated. This finding supports the notion that affective touch may play an important role in early brain development in humans. Further studies including additional tactile stimuli and longitudinal designs are required to assess the specificity of the responses to socio-affective tactile stimulation and implications for child development.

## Conflict of Interest

None.
